# Cancer registration for cancer control in Latin America: a status and progress report

**DOI:** 10.26633/RPSP.2017.2

**Published:** 2017-02-08

**Authors:** Marion Piñeros, M. Graciela Abriata, Les Mery, Freddie Bray

**Affiliations:** 1 Cancer Surveillance Section International Agency for Research on Cancer Lyon France; 2 Instituto Nacional del Cáncer Buenos Aires Argentina

**Keywords:** Neoplasms, surveillance, registries, health planning technical assistance, Latin America, Neoplasias, vigilancia, sistema de registros, asistencia técnica a la planificación en salud, América Latina

## Abstract

Cancer incidence by type has been included as a core indicator in the World Health Organization (WHO) Global Monitoring Framework for the Prevention and Control of Noncommunicable Diseases. The Global Initiative for Cancer Registry Development (GICR), coordinated by the International Agency for Research on Cancer (IARC), supports low- and middle-income countries to reduce disparities in cancer information for cancer control by increasing the coverage and quality of cancer registration. A baseline assessment has been performed at the IARC Regional Hub for Latin America using secondary and public information sources. Countries have been categorized according to the following criteria for population-based cancer registries (PBCRs): 1) “has no established PBCR (but some registration activity),” 2) “has established PBCR(s) but none of high-quality,” and 3) “has established, high-quality PBCR(s) (regional or national).” Currently, in LatinAmerica, most countries have cancer control plans in place; PBCRs cover approximately20% of the region’s population, though only 7% are deemed as having high-quality information. No information is available on the extent of use of the information generated by PBCRs for cancer control purposes. Though there are important advances in cancer registration in the region, there is still much to be done. This report also outlines key elementsfor improving cancer surveillance in the region, including 1) involvement of local stakeholders and experts, 2) integration of cancer registries into existing surveillance systems(accounting for the complexities and particularities of cancer surveillance), 3) improvementin data availability and quality, 4) enhanced communication and dissemination, and 5) better linkages between cancer registries and cancer planning and cancer research.

## RESUMEN

La incidencia del cáncer por tipo se ha incluido como indicador central del Marco mundial de vigilancia integral para la prevención y el control de las enfermedades no transmisibles de la Organización Mundial de la Salud (OMS). La Iniciativa Mundial para el Desarrollo de Registros de Cáncer (GICR por su sigla en inglés), bajo la coordinación del Centro Internacional de Investigaciones sobre el Cáncer (CIIC), brinda apoyo a los países de ingresos bajos y medianos para reducir las disparidades de información sobre el cáncer para el control de esta enfermedad mediante el aumento de la cobertura y la calidad de los registros de cáncer. El Núcleo Regional del CIIC para América Latina (Buenos Aires) realizó una evaluación de valores de referencia y comparación usando fuentes secundarias y de información pública. Los países se han clasificado según los siguientes criterios relativos a los registros de cáncer basados en la población: 1) “no tiene ningún registro”; 2) “ha establecido uno o varios registros, pero ninguno es de alta calidad”; y 3) “tiene un registro de alta calidad (regional o nacional)”. En América Latina, la mayor parte de los países ya han implantado planes de control del cáncer; los registros cubren aproximadamente 20% de la población de la región, aunque se considera que solo 7% tiene información de alta calidad. No hay ninguna información disponible sobre el grado de utilización de la información generada por estos registros para fines de control del cáncer. Si bien se observan adelantos importantes en cuanto a los registros de cáncer en la región, hay todavía mucho por hacer. En este informe también se describen los elementos principales para mejorar la vigilancia del cáncer en la región como: 1) la participación de interesados directos y expertos locales; 2) la integración de los registros de cáncer a los sistemas de vigilancia existentes (para tomar en cuenta las complejidades y particularidades de la vigilancia del cáncer); 3) el mejoramiento de la disponibilidad y la calidad de los datos; 4) el mejoramiento de la comunicación y la difusión; y 5) mejores vínculos entre los registros de cáncer y la planificación e investigación en materia de cáncer.

Cancer is a leading cause of morbidity and mortality worldwide. The estimated 14.1 million new cases of cancer and 8.2 million cancer deaths in 2012 are predicted to rise to over 22 million new cases and 14 million deaths by 2030, with higher proportional increases projected to occur in countries with “low” or “medium” scores on the Human Development Index (HDI) (1).

Long-term recommendations for tackling the rising cancer burden include implementation of national cancer control plans (NCCPs) (2), using high-quality cancer data obtained from population-based cancer registries (PBCRs), a cornerstone for cancer planning, monitoring, and evaluation (3). At the global level, countries have pledged to achieve a 25% reduction in mortality from noncommunicable diseases (NCDs) by 2025 (4) and (among other commitments) collect cancer incidence data by type of cancer, a core indicator of progress within the World Health Organization (WHO) Global Monitoring Framework for the Prevention and Control of Noncommunicable Diseases (5). A responsibility thus rests with governments to support the planning and developing of PBCRs to drive cancer policy.

There is a clear inequity in the coverage of cancer registration worldwide: high-quality cancer registration covers only 4%, 8%, and 7% of the populations in Africa, Asia, and Latin America respectively, while the equivalent coverage is 83% in North America and 32% in Europe (6). In 2011 the Global Initiative for Cancer Registry Development (GICR) was launched by the International Agency for Research on Cancer (IARC) (Lyon, France) to reduce these disparities by supporting low- and middle- income countries to increase the coverage and quality of their cancer registries (7). The emphasis within GICR is on building local and sustainable infrastructure through the establishment of regional centers of expertise (IARC Regional Hubs).

In Latin America, the IARC Regional Hub was established in 2013 coordinated by the National Cancer Institute in Buenos Aires, Argentina, alongside a supportive regional network of collaborators, to provide coverage for Spanishand Portuguese-speaking countries in Central America, the Caribbean, and South America. The need for cancer control in Latin America has received significant attention, with specific recommendations to increase investment in cancer registration (8); these have been recently followed-up in the form of a case study analyzing the development of cancer registration in one of the countries as part of a larger political commitment to cancer control (9, 10).

This report provides a status of cancer burden and registration in Latin America and outlines key elements for improving cancer surveillance in the region, including the need for 1) involvement of local stakeholders and experts, 2) integration of cancer registries into existing surveillance systems (accounting for the complexities and particularities of cancer surveillance), 3) improvement in data availability and quality, 4) enhanced communication and dissemination, and 5) better linkages between cancer registries and cancer planning and cancer research.

## Scale and profile of cancer in 2012 and projections of cancer burden by 2030

The total population of the 20 countries comprising the IARC Regional Hub for Latin America was approximately 600 million, in 2013 (11), representing about 9% of the global population (Table 1). Brazil and Mexico account for one-third of the Regional Hub population, with more than 200 million and 120 million inhabitants respectively. Three of the countries in the IARC Regional Hub have a “very high” score on the HDI; 11 have a “high” score and six have a “medium” score; the countries in the region with the lowest scores are Honduras and Nicaragua (12).

Overall, 1 million new cancer cases and 550 000 deaths are estimated to occur annually in Latin America (13). By 2030, the number of new cancer cases and deaths is expected to increase by 67%, with forecasted rises ranging from 23% in Uruguay to 92% in Costa Rica (13).

**TABLE 1.** Cancer burden and cancer control plans, by subregion and country, Latin America

		Cancer burden (2012)				Cancer control plans (2014)		
Subregion/country	Population (2013) (in thousands)	Incidence		Mortality		As part of NCD^b^ plan	National cancer control plan	For specific cancers
		Cases	ASR^a^ (per 100 000)	Deaths	ASR (per 100 000)			
South America								
Argentina	41 446	115 162	216.7	66 433	115.1	x		x
Bolivia (Plurinational State of)	10 671	11 286	143.9	6 939	90.9	x		x
Brazil	200 362	437 592	205.5	224 694	103.7	x		
Colombia	48 321	71 442	160.6	37 894	85.0	x	2012–2021	
Chile	17 620	40 414	175.7	25 049	103.0	x		x
Ecuador	15 738	23 360	164.5	1 384	94.5	x		
Paraguay	6 802	8 139	147.5	5 007	91.6	x		
Peru	30 376	42 846	154.5	26 165	92.1		2012–2015	
Uruguay	3 407	13 357	251.0	8 661	144.8	x	2005–2009	x
Venezuela (Bolivarian Republic of)	30 405	41 846	150.0	23 498	85.6	x		
Central America and the Caribbean									
Costa Rica	4 872	8 948	179.3	4 370	84.9	x		
Cuba	11 266	39 410	218.0	24 286	123.8	x		
Dominican Republic	10 404	14 680	153.4	9 046	90.5	x		
El Salvador	6 340	9 025	153.4	5 926	95.0	x	2011–2017	
Guatemala	15 468	13 271	130.4	9 871	96.4	x		
Honduras	8 098	7 431	131.2	5 050	90.3	x	2009–2013	
Mexico	122 332	147 985	131.5	78 719	68.9	UD^c^		
Nicaragua	6 080	5 129	114.4	3 568	80.9	UD		
Panama	3 864	5 415	148.4	2 942	79.1	x	2010–2015	
Puerto Rico	3 688	11 822	211.1	4 678	71.1	x	2008–2012	

***Source:*** Prepared by the authors using data from the following sources: population (11); cancer burden 2012 (13); cancer control plans and plans for specific cancers (14–18).

a ASR: age-standardized rate.

b NCD: noncommunicable disease.

c UD: under development.

**FIGURE 1.**
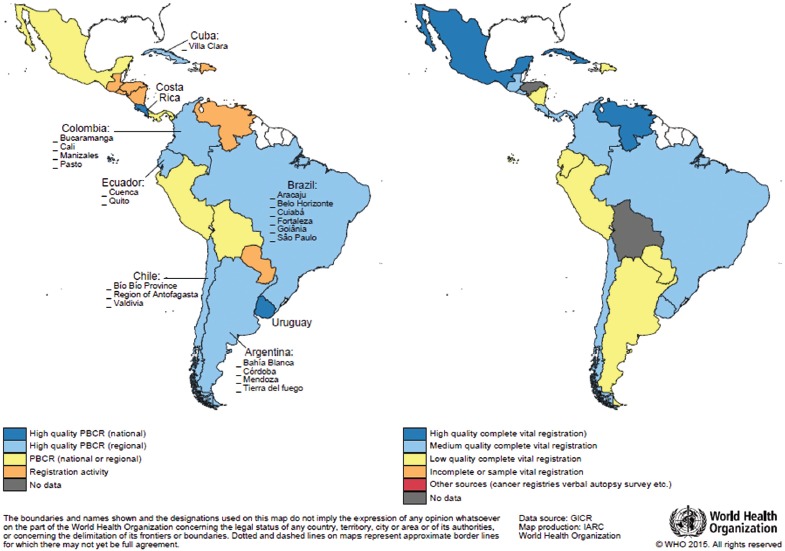
Status of 1) population-based cancer registries (PBCRs) (left) and 2) vital registration systems (right), Latin America, 2014

***Source:*** prepared by IARC/WHO using references (6, 22). Reprinted with permission.

Cancer incidence tends to be slightly lower in Central American countries, with age-standardized rates (ASRs) (per 100 000) ranging from 130 (in Guatemala) to 218 (in Cuba), compared to South America, where rates range from 143 (in Bolivia) to 251 (in Uruguay) (Table 1). Overall incidence in both subregions (134 and 191 respectively) is lower than that in greater Europe (ASR: 255) and North America (ASR: 316). While the corresponding ASRs for mortality in the region range from 71 (in Puerto Rico) to 144 (in Uruguay) (Table 1), they are, in most countries, similar to those in Europe (ASR: 113) and North America (ASR: 106) (13). As a result, mortality incidence ratios are high in Latin America, particularly for cancers that are associated with early detection, such as female breast cancer.

For Central and South America combined, the most frequent cancer among males is prostate (27.6% of all male cases), followed by lung (9.6%), colorectal (8%), and stomach cancer (7.1%). Among females, breast cancer is most common (27% of all female cases), followed by cervical (12.3%), colorectal (7.7%), and lung cancer (5.5%) (13). Trends in most countries for the leading cancers in the region reveal rising ASRs for mortality from colorectal cancer in males and females and from lung and breast cancer in females (19). Declining trends are observed for stomach cancer in both sexes, as well as lung cancer (in men) and cervical cancer. The apparent transition toward a more Westernized cancer profile is evident but remains only partial, given the high burden of stomach and cervical cancer in many countries in the region (19, 20).

## Current status of cancer surveillance data and cancer control planning

The availability of cancer incidence and mortality data from established surveillance activities (21) for the region is shown in Figure 1. Every country in the IARC Regional Hub for Latin America has some form of cancer registration; nevertheless, there are more countries with high-quality PBCRs^3^ (and thus high-quality incidence data) in South America than in Central America.

Vital statistics systems are in place in all 20 countries comprising the IARC Regional Hub, with the coverage of civil registration by cause of death ranging from around 60% to almost 100%, except in Bolivia and Honduras, where no information (by cause) is currently available (Figure 1) (22).

The majority of countries in the IARC Regional Hub have national cancer control plans in place, most of which are integrated into broader NCD plans and were developed rather recently. In addition, 11 countries have developed either comprehensive cancer control plans or plans for specific cancers (14–18). Table 1 provides data on these cancer plans, and the cancer burden, by country. Unfortunately, no comparable information on financing or implementation (or the impact) of cancer control activities is available at the regional level. Similarly, there is very limited knowledge of the extent to which cancer planning includes cancer surveillance as a central component.

## Cancer registration: availability, coverage, representativeness, and quality

**FIGURE 2.**
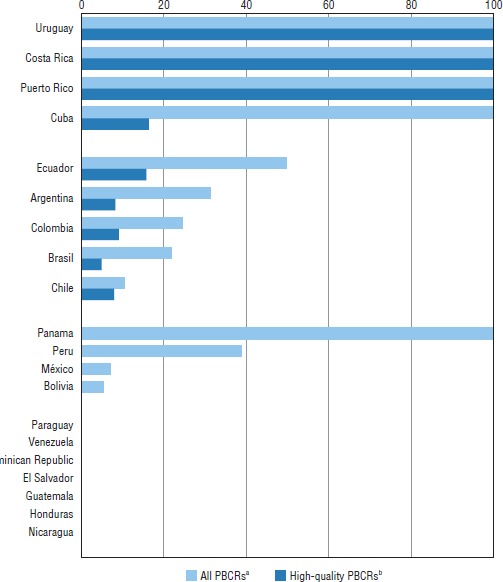
Population covered (%) by population-based cancer registries (PBCRs), by country and PBCR quality, Latin America, 2014

***Source:*** Prepared by the authors based on the study results.

^a^ All PBCRs according to the study results.

^b^ PBCRs with data included in the International Agency for Cancer Research (IARC) publication *Cancer Incidence in Five Continents* (CI5) (volume X) (6).

The initial development of PBCRs in the region began in the 1950s, with the cancer registries in Puerto Rico (1951) (23), Cali (1962) (24), and São Paulo (1963) (25). At present an estimated 91 PBCRs are operating throughout Latin America (69 in South America and 22 in Central America). Five countries have national cancer registries; the proportion of the population covered by the existing cancer registries in the region is a little more than one-fifth (20.3%), with high-quality information coverage (i.e., inclusion in the CI5) estimated at 7.1%. Based on available information on the coverage and quality of the existing PBCRs, they can be categorized as follows (see also Figure 2): 1) countries with no established PBCR but some registration activity; 2) countries with an established PBCR but no high-quality PBCR; 3) countries with regional PBCRs, some of which are high-quality PBCRs; and 4) countries with national high-quality cancer registration coverage. In the third group of countries, the population covered by high-quality PBCR reaches around 10% (Figure 2). It is noteworthy that three countries have cancer estimates at a subnational level based on their PBCR(s) (26–28).

**FIGURE 3.**
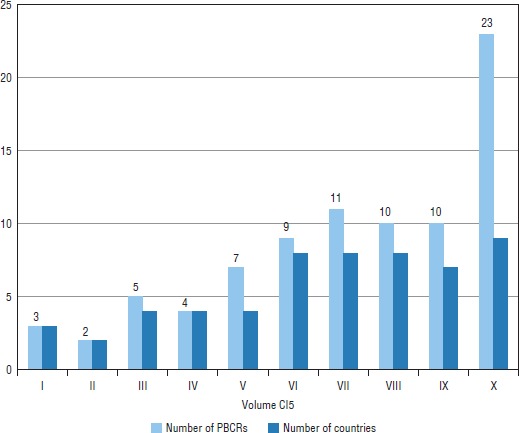
Coverage of population-based cancer registries (PBCRs) in volumes I–X of the International Agency for Research on Cancer (IARC) publication *Cancer Incidence in Five Continents* (CI5), Latin America, 2014

***Source***: Prepared by the authors based on data from the IARC (http://ci5.iarc.fr).

The evolution of the inclusion of the information from Latin American PBCRs in the C15 publication is shown in Figure 3. While there has been an increase in the number of registries, particularly in the most recent volume (X), the number of countries has remained almost stable over the past three editions (VIII–X), indicating that the increase in registries that accomplish high-quality standards occurs mainly in the same countries. In volume X, 13 registries were added compared to the previous volume, the majority of which were from Argentina, Brazil, Chile, and Colombia (6).

## Considerations for improving cancer surveillance in the region

**Involving local stakeholders and experts.** Despite their relatively low coverage, many of the existing high-quality PBCRs in Latin America have a long history of operation, and staff with extensive experience and expertise. Fostering and using this capacity is a key strategy for improving cancer surveillance. In the region, the transfer of technical knowledge between countries can be developed among regional partners with sufficient mutual trust, a crucial component of technical assistance (29). Building a roster of supportive experts in different domains is one example of aid through registry mentorship that seeks to improve specific aspects of cancer registration and use of the data. Within the region, designated collaborating centers are providing the platform to develop twinning programs and specific roles for learned institutions within countries.

It is equally important to develop linkages with other bodies already involved with cancer surveillance in the region. These include the Pan American Health Organization (PAHO); the Ibero-American Network of Cancer Epidemiology and Information Systems (REDEPICAN); and the Network of National Cancer Institutes (*Red de Institutos Nacionales de Cáncer*, RINC). Each of these entities 1) has established networks (e.g., the RINC’s Operative Group for Cancer Registration); 2) has longstanding experience in providing consultations with the registries (e.g., REDEPICAN) (30); and/ or 3) provides assistance in other ways (e.g., PAHO’s development of Cancer Country Profiles) (31). In the operative model for the IARC Regional Hub implemented in Latin America, the participation of experts and the aforementioned organizations is secured through agreements with designated collaborating centers to engage regional experts in different activities, including teaching and site visits and inclusion in the Regional Hub Advisory Committee.

**Integrating cancer registries into existing surveillance systems.** One issue particularly relevant to the region and common to many countries in epidemiological transition is the need for the provision of clear concepts to health care professionals and decision-makers regarding differences in surveillance for different diseases, specifically the distinction between acute communicable diseases versus NCDs, and within NCDs, the specifics of cancer surveillance.

The surveillance of communicable diseases—with a focus on interruption or containment of transmission and potential expansion of infection—is an often well-established activity within health systems supported by mandatory notification in the region. Surveillance of the more recently introduced NCDs has until now focused mostly on risk factor surveillance and comparable periodic surveys (32).

Surveillance of cancer incidence is quite different, however, because of the complexity of cancer, which has numerous distinct entities that vary biologically, clinically, and epidemiologically. Cancer registration (yielding cancer incidence and survival information) can be seen as a strategy that is complementary to yet distinct from NCD surveillance. It requires not only a system for classification and coding of neoplasms but also a clear definition of what constitutes a cancer case; the definition of the date of incidence; and the rules for dealing with multiple primary cancers, including the need to differentiate a new case of a primary cancer from the extension, recurrence, or metastasis of an existing one (21). Accurate, timely, and complete case ascertainment is essential in population-based cancer registration. This requires trained personnel and adherence to international standards for registering cancer cases (33).

A complete understanding of differences in cancer surveillance relative to other diseases is particularly important in the Latin American region where, supported by the experience of communicable disease surveillance, many Ministries of Health have promoted cancer surveillance systems based on cancer notifications that aim to offer complete and national coverage. These initiatives tend to have been put in place without direct assessments of data quality to validate the potential for collecting the information as well as its quality. Reliance solely on passive (electronic) registration practices does not seem to be an appropriate strategy to obtain complete and accurate incidence data. Furthermore, for the purposes of cancer control and cancer research, the benefits of increasing coverage of PBCRs are usually sufficiently high when there are one or more wellfunctioning regional (subnational) PBCRs that can be considered, collectively, to be nationally representative; in large populations, national cancer registries are usually neither feasible nor cost-effective (21). With these considerations, in addition to the cost and sustainability of nationwide projects, countries should aim to establish regional PBCRs that comply with the documented requirements (21).

## Getting better registry data: enhancing availability and quality

Along with strong political will and commitment, efforts to improve cancer registration coverage require a clear understanding of cancer surveillance, including the definition of a “representative” geographic area (“catchment area”) to be covered by the cancer registry. There is a cluster of countries in Central America, with “medium” HDI scores, without established PBCRs, where the populations are relatively small, ranging from 4 to 14 million. In advancing change, one key issue for these countries is the definition of the PBCR catchment areas that support cancer control planning (i.e., those that would help ensure registry sustainability, technical feasibility, and the possibility of extrapolating the data that are generated beyond the catchment area) (21). These considerations and guidelines, provided by IARC, also apply to countries that have existing regional cancer registries but wish to augment surveillance with additional registries to increase coverage and representativeness. In this situation, a key consideration is the inclusion of additional, well-defined regions that are demographically or geographically distinct.

In many Latin American countries, childhood cancer mortality rates are among the highest in the world (22), a fact that has attracted international collaboration and led to the establishment of twinning programs to improve pediatric cancer care (34, 35). These initiatives have been accompanied in most cases by the development of hospital-based, outcome-oriented information systems (36) that, given the relatively small numbers of pediatric cancer cases, are of enormous value in efforts to establish central cancer registries and provide information to PBCRs.

Ensuring the good quality of the data produced by the cancer registries, with the basic elements of well-described comparability, validity, timeliness, and completeness, is essential (33, 37). Improving data quality requires staff training, consultancies, recommendations, and follow-up tailored to a country’s needs. One priority of the GICR in the region is to work with cancer registries that have submitted data to the most recent volume of the CI5 (volume X) but thus far are not included in it. Another focus of the work will be the establishment of regular training courses, and the development of educational materials in Spanish, including content for webinars, e-learning, and discussion forums that enables registry personnel to exchange information and respond to queries.

Activities to improve registry data require continued and regular communication between the registries and the providers of the technical assistance, including the IARC Regional Hub coordinating center, the collaborating centers, and the IARC. As Le et al. have established, technical assistance is a mix of content-driven and relationship-based approaches, with the first focusing on information transfer and referral and the second focusing on facilitation of systems change within a given context (38).

## Making cancer data count: linking registries to cancer planning and research

Dissemination of data is one of the central activities of all cancer registries, and high-quality publications relevant to cancer control help support their sustainability. While the key elements and general outline of a registry report are well defined (21), it is important to disseminate information using formats tailored to different audiences. The dissemination of information for cancer control planning purposes must kindle relationships with decision-makers, practitioners, and policymakers in real time. Decision-makers often prefer shorter reports and the development of interactive knowledge transfer strategies (39, 40). There is at present substantial variability in the structure and content of registry reports in the region, and technical guidance is needed to standardize reports for comparability purposes while ensuring the enhanced credibility and visibility of the registries in the eyes of key stakeholders.

One priority for the IARC Regional Hub is to promote cancer research by identifying topics of common interest to the community that directly contribute to cancer control and thus foster collaborative cancer research across countries. Although the number of published studies in the field of epidemiology and public health has increased significantly in the region (41, 42), dissemination remains limited compared to many countries with a “very high” HDI score (43). Much of the scientific productivity is in Spanish, which has increasing visibility in regional databases (44, 45) but is limited in the international context, particularly if the notably low-impact factor of Latin American publications is considered (46). Further, there is evidence that much of the postgraduate epidemiology training in the region is oriented toward communicable diseases (41). Cancer registries offer the possibility for public health and epidemiology training in cancer within the context of NCDs, which can strengthen relationships with universities and enhance scientific productivity. The IARC project “Cancer in Central and South America,” and its associated publications (47), along with other recent publications (48), are valuable instruments for identifying future research and establishing a cancer research plan for the Regional Hub.

Finally, although cancer is a major cause of premature death, it has also become (for certain cancer types) a chronic condition, especially in countries transitioning toward very high levels of human development, a change that has been linked to earlier diagnosis, better management, and therapy. Therefore, along with incidence (and mortality), registries can provide additional essential population-based indicators for assessing and quantifying efforts to reduce the burden of, and suffering from, the disease. These include the proportion surviving a cancer diagnosis, years of life lost due to cancer death, and, among cancer survivors, years of life with disability following initial diagnosis.

## Conclusions

Important advances have been made in cancer registration in Latin America, with improvements in quality, coverage, and use providing a necessary boost to the promotion of their centrality in cancer control planning. However, much remains to be done to fill gaps in cancer surveillance in the region. The establishment of PBCRs is a clear priority in Central American countries, whereas South American registries would benefit from improvements in data quality and a greater use of the most valuable data. With the involvement of local stakeholders and experts, the integration of cancer registries into existing surveillance systems, enhanced communication, and sufficient dissemination of the results, as well as better linkages between cancer registries and programs of cancer control and research, registries could flourish in every country in the region. The IARC Regional Hub, now implemented, provides the mechanism for expanding the required technical support, training, advocacy, and networking throughout Latin America.

## Acknowledgments.

The authors gratefully acknowledge Patricia Cueva (Quito Cancer Registry), Yaima Galan (Cuba National Cancer Registry), Marise Rebelo (Cancer Surveillance at the National Cancer Institute of Brazil), and Clelia Vallebuona (Epidemiology Department, Chile Ministry of Health), who reviewed the information on the existing PBCRs in their respective countries. They also thank Mathieu Laversanne (IARC), who provided Figure 1.

## Disclaimer.

Authors hold sole responsibility for the views expressed in the manuscript, which may not necessarily reflect the opinion or policy of the RPSP/ PAJPH or the Pan American Health Organization (PAHO).

## Footnotes

3 Defined as PBCRs that are included in the latest volume (X) of IARC’s comparative quinquennial publication, Cancer Incidence in Five Continents (CI5) (6).
